# Insights into Rotational and Translational Dynamics in Mixtures of Ethylene Glycol and Choline Chloride Using Nuclear Magnetic Resonance Techniques

**DOI:** 10.1021/acs.jpcb.6c01832

**Published:** 2026-05-28

**Authors:** Carla C. Fraenza, Ramez A. Elgammal, Thomas A. Zawodzinski, Steven G. Greenbaum

**Affiliations:** † Department of Physics and Astronomy, 5924 Hunter College of CUNY, New York, New York 10065, United States; ‡ Department of Chemical and Biomolecular Engineering, 4292 University of Tennessee-Knoxville, Knoxville, Tennessee 37996, United States

## Abstract

This work examines molecular dynamics and interactions in ethylene glycol–choline chloride (EG–ChCl) mixtures across 0–33 mol % ChCl, spanning the true eutectic region near 17–20 mol % and the commonly used 1:2 formulation. We combine pulsed-field-gradient (PFG) diffusion, fast-field-cycling (FFC) relaxometry, temperature-dependent ^13^C *T*_1_, and nuclear Overhauser effect spectroscopy (NOESY) to disentangle local from macroscopic dynamics. PFG and FFC show that both translational and average rotational motions largely track the strong increase in viscosity with ChCl content, with ethylene glycol consistently diffusing faster than the choline cation and no global dynamical anomaly at the eutectic composition. More subtle, site-specific composition effects nevertheless emerge. The ratio of the diffusion coefficient of the hydroxyl group of choline to the diffusion coefficient of the methyl group of choline displays a shallow minimum in the 17–25 mol % region, indicating a modest change in how the hydroxyl-bearing end of choline samples the underlying translational motion relative to the methyl groups. ^13^C *T*_1_ analysis shows that rotational correlation times at 25 °C generally increase with ChCl, reflecting viscosity-coupled slowing, while the CH_2_–N_α_ site exhibits a small but reproducible deviation from this monotonic trend near the eutectic. NOESY spectra at similar compositions reveal enhanced cross-relaxation between EG and choline protons, consistent with increased headgroup–solvent contact density rather than a wholesale structural rearrangement. Overall, our multitechnique study demonstrates that EG–ChCl dynamics are predominantly viscosity-dominated, with the eutectic region acting as a subtle dynamical crossover where specific choline segments become maximally coupled to the hydrogen-bond network. These insights refine the structure–dynamics picture of choline-chloride DESs and provide practical guidance for tuning composition in electrochemical, separation, and catalytic applications.

## Graphical abstract

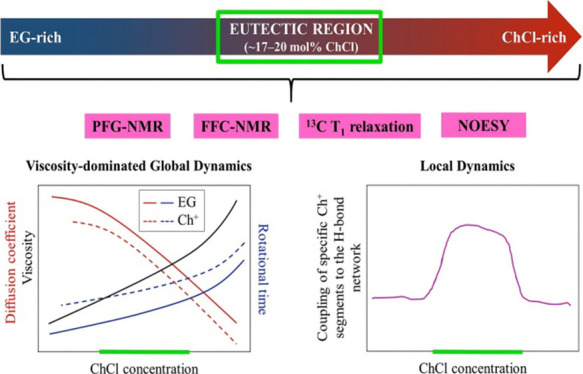

## 1 Introduction

Deep eutectic solvents (DESs) are commonly described as hydrogen-bonded mixtures of a hydrogen bond donor (HBD) and a hydrogen bond acceptor (HBA), often a quaternary ammonium salt, whose melting points lie well below those of their individual components.^1–5^ However, recent critical assessments have highlighted that the term “DES” has been applied very broadly, often without sufficient thermodynamic characterization. In particular, it has become clear that one must distinguish between (i) truly *deep* eutectic solvents that show large negative deviations from ideal solid–liquid equilibrium behavior, (ii) nearly ideal eutectic solvents, and (iii) low-transition-temperature mixtures that are metastable liquids. These classifications are grounded in excess Gibbs energies of mixing and eutectic depth rather than solely in low melting points.^6,7^ This more stringent view is especially important for choline chloride based systems, which are often promoted as “green” and benign yet may not always fulfill the expected criteria for sustainability or nontoxicity.^5,8,9^

Among the most widely studied systems is the choline chloride–ethylene glycol mixture, often referred to as “ethaline” at a 1:2 molar ratio.^10–12^ Historically, this 33 mol % ChCl mixture has been treated as the eutectic and as a paradigmatic DES. More recent solid–liquid equilibrium measurements paint a different picture. Agieienko and Buchner showed that the phase diagram of EG–ChCl is very close to that of an ideal binary mixture, with a true eutectic located near 17 mol % ChCl and a melting point only about 16 K below that of pure ethylene glycol.^13–15^ Based on this prior work, the samples remain liquid even up to the highest ChCl (33 mol %) concentration. Thus, no solidification artifacts are expected. Complementary calorimetric and modeling studies have introduced an empirical descriptor of “eutectic depth” and demonstrated that, by this metric, EG–ChCl is only weakly deep compared to other choline chloride based mixtures; its excess Gibbs energy of mixing is modest and largely entropy dominated.^7,16^

These thermodynamic findings have direct implications for the expected dynamical behavior of the liquid. In a truly “deep” eutectic system characterized by large negative deviations from ideality, one expects the formation of strong, long-lived supramolecular complexes (e.g., discrete hydrogen-bonded clusters) that can lead to dynamical arrest or pronounced decoupling from bulk viscosity. Conversely, the near-ideality observed in EG–ChCl implies that interactions between unlike species (EG–choline) are energetically similar to interactions between like species. Consequently, one would not anticipate singular “lock-up” or sharp structural transitions at the eutectic composition. Instead, a first expectation is that transport properties should evolve continuously with composition and broadly scale with the macroscopic viscosity, which increases smoothly as the mixture transitions from a molecular solvent to a salt-rich fluid. This view is consistent with recent electrical conductivity data, which show a broad maximum rather than a sharp peak around 20–25 mol % ChCl.^13,17–19^ That maximum has been interpreted using hole theory as a crossover from an electrolyte-solution regime at low salt content, where increasing ChCl primarily increases the number of mobile ionic species, to a salt-rich regime at higher fractions, where the supply of free solvent molecules able to solvate ions becomes limited and charge transport proceeds increasingly via the formation and diffusion of ion-sized voids.^17^ In this picture, the near-eutectic region marks a change in transport mechanism rather than a unique structural state, and any “special” behavior at this composition is expected to be observable-dependent, reflecting a crossover between solvent-rich and salt-rich transport regimes rather than a single “canonical” ratio.

The microscopic structure and dynamics of EG–ChCl mixtures are central to interpreting these macroscopic trends. Ethylene glycol forms an extended hydrogen-bond network in the neat liquid, and the addition of ChCl disrupts this network and introduces chloride-centered coordination motifs. Molecular simulations and spectroscopic studies on choline chloride based mixtures have shown that these liquids are microheterogeneous, with nanometer-scale domains enriched in ionic or HBD species and with solvation environments that depend sensitively on both composition and probe identity.^7,12,19–22^ Recent nuclear magnetic resonance (NMR) studies on related systems such as choline chloride–glycerol (glyceline) have shown that incorporating ChCl induces dynamic heterogeneity and additional relaxation modes, with the balance between ionic and HBD components strongly influencing which species experiences the greater friction.^23^ Collectively, this emphasizes that translational and rotational dynamics in DESs can decouple significantly from bulk viscosity and from each other, with neutral, cationic, and anionic species experiencing very different local frictions within the same macroscopic liquid and that specific compositions, including but not limited to the eutectic, may act as dynamical crossovers where the roles of cation and HBD interchange or become more strongly coupled.

Fluorescence anisotropy measurements on EG–ChCl illustrate this complexity: neutral and cationic dyes can reorient faster than predicted from macroscopic viscosity, whereas anionic probes remain tightly coupled to the slow, ionic hydrogen-bond network and follow Debye–Stokes–Einstein behavior.^24^ Relaxation studies on related systems reinforce the role of the HBD in setting the local friction.^21^ In “ethaline” the ethylene glycol reorients faster than choline, while in the choline chloride–glycerol mixture (“glyceline”) the opposite trend is observed, underscoring how the identity and hydrogen-bonding propensity of the HBD determine which component is more strongly hindered.^23,25^ These results collectively suggest that any apparent eutectic “anomaly” in EG–ChCl is likely to manifest not as a universal feature, but rather through specific changes in the local environment of selected molecular sites.

In this context, EG–ChCl is thermodynamically closer to an ordinary eutectic or low-transition-temperature mixture than to strongly nonideal DESs such as reline, but it remains an important model system for probing how microscopic structure and dynamics evolve across composition in hydrogen-bond–rich liquids. In the present work we combine several complementary NMR techniques to address this question. Pulsed field gradient (PFG) NMR provides direct measurements of the self-diffusion coefficients of ethylene glycol and choline as a function of composition and temperature. Fast field cycling (FFC) NMR relaxometry measures the dispersion of the longitudinal relaxation rate over a wide frequency range and is sensitive to both fast and slow rotational motions. These approaches are complemented by temperature-dependent ^13^C longitudinal relaxation experiments at fixed field, which allow us to extract effective rotational correlation times for individual carbon sites on both choline and ethylene glycol, and by two-dimensional NOESY experiments that report on short-range spatial proximities and site-specific exchange processes. Together, these data provide a coherent picture of how hydrogen bonding, local friction, and proton exchange evolve across the EG–ChCl composition space, and they allow us to identify which aspects of molecular dynamics are simply viscosity-dominated and which show subtle but reproducible sensitivity to the near-eutectic regime.

## 2 Experimental Section

### 2.1 Materials and Sample Preparation

Ethylene glycol (EG) and choline chloride (ChCl) were purchased from ThermoFisher Scientific and Sigma-Aldrich, respectively, both at ≥99% purity. Deuterated EG (EG-d6) and ChCl (Ch-d9Cl) were obtained from CDN ISOTOPES at ≥98% purity. The purpose of using deuterated reagents is to isolate the ^1^H nuclei dynamics on EG or choline when the low-resolution FFC-NMR technique is used; we do not consider possible changes in H-bonding strength or other properties as a result of deuteration. The reagents were kept under an argon atmosphere in a glovebox and used as received. Different amounts of ChCl (or Ch-d9Cl) were added to EG (or EG-d6), namely 5, 10, 17, 20, 25, and 33 mol %, including the eutectic concentration (15–20 mol % ChCl). The two components were mixed and heated at 80 °C using a magnetic stirrer hot plate until a clear liquid was formed and then hermetically sealed into NMR tubes before usage. The molecular structure of EG, EG-d6, ChCl, and Ch-d9Cl are shown in Figure 1.

**Figure 1:**
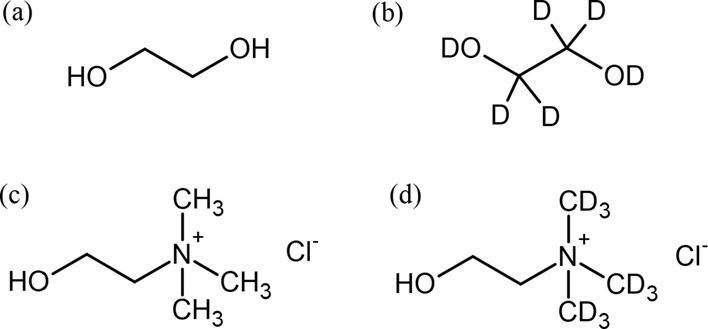
Molecular structure of (a) ethylene glycol, (b) ethylene glycol-d6, (c) choline chloride, and (c) choline-d9 chloride.

### 2.2 Diffusion Measurements Using Pulsed Field Gradient (PFG) NMR

Diffusion experiments were performed with a 400 MHz Bruker spectrometer operating at a magnetic field of 9.4 T. ^1^H NMR spectrum showed well-resolved peaks for each component of our samples. Therefore, EG and choline (Ch^+^) self-diffusion coefficients were measured independently via the PFG-NMR stimulated echo sequence, at variable temperatures from 25 to 65 °C. The self-diffusion coefficients (*D*) were calculated by fitting the decay of the echo signal with the Stejskal-Tanner equation.^26^ The equation is defined as follows: *I* = *I*_0_ exp­[−(δ*G*γ)^2^ (Δ−δ/3) *D*)], where *I* represents the amplitude of the attenuated echo signal, *I*_0_ is the initial intensity, δ is the gradient pulse duration, *G* is the gradient strength, γ is the hydrogen gyromagnetic ratio, and Δ is the diffusion time. In our experiments, *G* took 16 different values following a linear increase and their values were in the range of 2–98% of a maximum strength of 50 G cm^–1^. δ and Δ were in the range of 3–6 ms and 0.1–0.5 s, respectively. Unfortunately, due to the large electric quadrupole moment of the ^35^Cl nucleus, the relaxation is too rapid for measuring chloride ion diffusion using PFG methods.

### 2.3 Dispersions of the Longitudinal Relaxation Rate by Fast Field Cycling (FFC) NMR Relaxometry

^1^H longitudinal relaxation rate (*R*_1_ = 1/*T*_1_) dispersions for EG and Ch^+^ were measured using a Spinmaster FFC2000 CDC Relaxometer (Stelar; Mede, Italy). FFC-NMR^27–29^ is a low-resolution technique; therefore EG-d6 and Ch-9Cl were used to isolate choline and EG dynamics, respectively. Using these isotopologues of EG and ChCl, the hydrogens present in choline and EG can be selectively studied without background from those present in EG and Ch^+^, respectively. *R*_1_ was measured using the standard prepolarized (PP) and nonpolarized (NP) sequences.^27^ The polarization magnetic field was 0.352 T (equivalent to 15.0 MHz for ^1^H Larmor frequency), and the acquisition magnetic field was 0.383 T (equivalent to 16.3 MHz for ^1^H Larmor frequency). The relaxation magnetic field took 20 different values distributed in a window ranging from 30 kHz to 15 MHz in ^1^H Larmor frequencies. The field slew rate was 13 MHz/ms and the switching time was 3 ms. At all relaxation frequencies, the magnetization evolution was monoexponential and could be fitted by a monoexponential function within acceptable errors. *R*_1_ experimental uncertainties in the relaxation profiles were calculated from these monoexponential fittings, which yielded values of typically less than 5%, which are typically covered by the size of the data points in the graphs. The sample temperature was controlled within ± 1 °C using a Stelar variable temperature controller (VTC). The relaxation profiles of samples without and with ChCl (10, 20, and 33 mol %) were measured at a temperature of −5 °C. The choice of this temperature was based on the observation of almost no dispersion of the relaxation profiles at higher temperatures. Relaxation dispersion curves were modeled by using a previously discussed methodology.^30^

### 2.4 Temperature-Dependent Single Frequency NMR Relaxation and Nuclear Overhauser Effect Spectroscopy (NOESY)

^1^H and ^13^C NMR data were recorded on a Bruker Avance III spectrometer operating at 400 MHz equipped with a 5 mm broadband probe. Samples were equilibrated at each set temperature for at least 1 h prior to data acquisition. Chemical shifts were referenced to TMS as an external standard. ^1^H spectra were acquired using a standard single-pulse experiment (90° flip angle, 20 ppm spectral width, 5 s relaxation delay). ^13^C spectra were acquired with a single-pulse experiment (90° flip angle, 250 ppm spectral width, 2 s relaxation delay). ^13^C longitudinal relaxation times *T*_1_ were measured using an inversion–recovery pulse sequence with inverse-gated proton decoupling and a 5 s relaxation delay. *T*_1_ values were obtained from monoexponential fits; the experimental uncertainties were less than 4%, smaller than the symbols used in the plots. Variable-temperature measurements were carried out from −45 to 65 °C. Because the ^13^C *T*_1_(T) curves for the ChCl–EG mixtures do not exhibit well-resolved minima within this window, the data at 25 °C lie on the fast-motion side of the dipolar relaxation dispersion. In this regime, 1/*T*_1_ scales approximately with the rotational correlation time τ_c_ for a given site, and effective τ_c_ values at 25 °C were estimated using the Solomon–Bloembergen heteronuclear dipolar treatment as a comparative measure of local reorientational dynamics across compositions.^31,32^ Phase-sensitive ^1^H–^1^H NOESY spectra were recorded using a gradient-selected NOESY sequence with mixing times between 50 and 800 ms; these experiments were used qualitatively to assess relative spatial proximities and exchange pathways.

## 3 Results and Discussion

A series of experiments was conducted to investigate how ChCl influences the dynamics of EG. First, we collected experimental data for pure EG. Then, we gradually increased the concentration of ChCl up to 33 mol %. It is important to note that this concentration exceeds the eutectic composition, which ranges from 15 to 20 mol % of ChCl.^13,33^

### 3.1 Diffusion Measurements

Self-diffusion coefficients of ethylene glycol (EG) and choline cation (Ch^+^) were determined separately using the ^1^H NMR spectrum signals of the −CH_2_ and methyl group (−CH_3_), respectively. The values of these coefficients at different ChCl concentrations and as a function of temperature are shown in Figures 2a,b. Experimental uncertainties were typically less than 4% and within the size of the data points. For temperatures lower than 55 °C, the diffusion coefficients of both molecules increase with temperature and decrease with concentration of ChCl, and this is consistent with the corresponding changes in viscosity.^17,34^ However, at 55 °C some of these coefficients start merging and a crossover effect is observed at 65 °C. For instance, at 65 °C the diffusivity of choline in the sample with 17 mol % ChCl (3 × 10^–10^ m^2^/s) is larger than its value at 10 mol % ChCl (2.5 × 10^–10^ m^2^/s). This is because the temperature dependence of viscosity also varies with the concentration of ChCl.^34^

**Figure 2:**
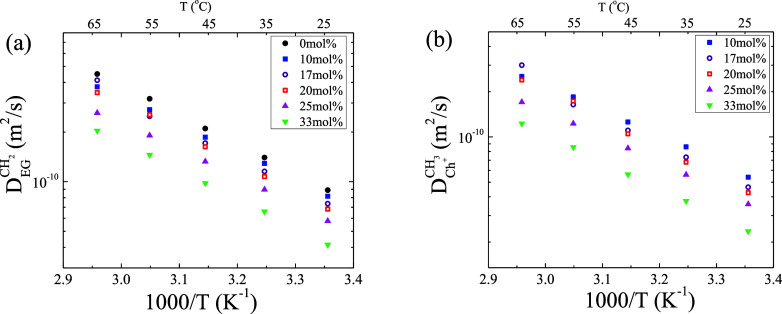
Temperature dependence of (a) EG (−CH_2_) and (b) choline (−CH_3_) self-diffusion coefficients at different ChCl concentrations. For both molecules, a crossover in the magnitude of *D* is observed at 65 °C.

At all temperatures and ChCl concentrations, *D*_EG_ is greater than *D*_Ch+_ due to the smaller molecular weight and charge neutrality of EG, as illustrated in Figure 3 at 25 °C. It is interesting to notice that the diffusion coefficients do not display any distinctive behavior across the eutectic concentration range (15–20 mol % ChCl), and only the viscosity effect is observed. Therefore, it appears that the presence of ChCl does not disturb the EG H-bonding network as it does in the case of glyceline.^23^ This is attributed to the fact that EG interacts with chloride and choline primarily through its two hydroxyl (−OH) groups,^12^ whereas glycerol interacts using its three −OH groups.^35^ Furthermore, it has been shown that the main interaction between the components of a polyol-ChCl mixture is the hydrogen bond interaction between the chloride ion (Cl^–^) of ChCl and the O–H group of the polyol, and its interaction strength decreases as the number of hydroxyl groups in the polyol decreases.^36^

**Figure 3:**
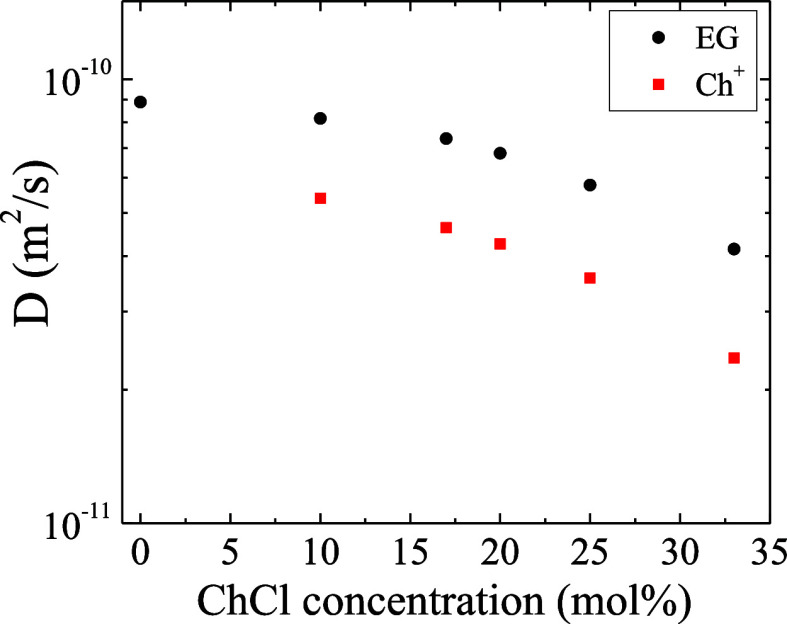
Diffusion coefficients of EG (−CH_2_) and choline (−CH_3_) as a function of ChCl concentration at 25 °C.

It is important to note that the highest ionic conductivity is achieved within the eutectic concentration range, as reported in the literature.^17,33^ This suggests that we would expect to see a peak in the diffusion coefficient values within this range (15–20 mol % ChCl). However, our experimental diffusion data do not exhibit any special features within the eutectic concentration range (Figure 3). One possible explanation for this discrepancy is that our PFG experiments only provide information on the translational movement of the positive ion (choline). Therefore, it is likely that the maximum conductivity is primarily attributed to the negative ion (chloride). This explanation is consistent with molecular dynamics simulations that revealed that Cl^–^ ions exhibit higher diffusion coefficients than Ch^+^ ions, indicating that Cl^–^ contributes more significantly to the overall ionic conductivity of the DES.^37^

Additionally, the NMR signals of the hydroxyl groups (−OH) of EG and choline were analyzed to determine the diffusion coefficients ${D_{\mathrm{EG}}}^{\mathrm{OH}}$ and ${D_{{\mathrm{Ch}}^{+}}}^{\mathrm{OH}}$ , respectively. In order to compare these coefficients with the values shown in Figure 2, the ratio of ${D_{\mathrm{EG}}}^{\mathrm{OH}}$ to ${D_{\mathrm{EG}}}^{{\mathrm{CH}}_{2}}$ and ${D_{{\mathrm{Ch}}^{+}}}^{\mathrm{OH}}$ to ${D_{{\mathrm{Ch}}^{+}}}^{{\mathrm{CH}}_{3}}$ were calculated. These ratios are presented as a function of ChCl concentration at different temperatures in Figure 4a,b, respectively. It is observed that ${D_{\mathrm{EG}}}^{\mathrm{OH}}$ / ${D_{\mathrm{EG}}}^{{\mathrm{CH}}_{2}}$ ≈ 1 but ${D_{{\mathrm{Ch}}^{+}}}^{\mathrm{OH}}$ / ${D_{{\mathrm{Ch}}^{+}}}^{{\mathrm{CH}}_{3}}$ > 1, which means that choline has two different diffusion values. This type of behavior is typical for molecules that experience hydrogen exchange between hydroxyl groups with other species and if this phenomenon becomes significant, a large difference in the diffusion coefficient values of the aliphatic and hydroxyl groups of the same molecule might be expected.^38,39^ This exchange appears to be slow enough to be observed at the time scales of our NMR experiments, which range from milliseconds to seconds. One possible explanation for this slow hydrogen exchange could be the relatively large distance between these −OH groups. They are separated by chloride ions that are involved in hydrogen-bond interactions with EG.^12,35^

**Figure 4:**
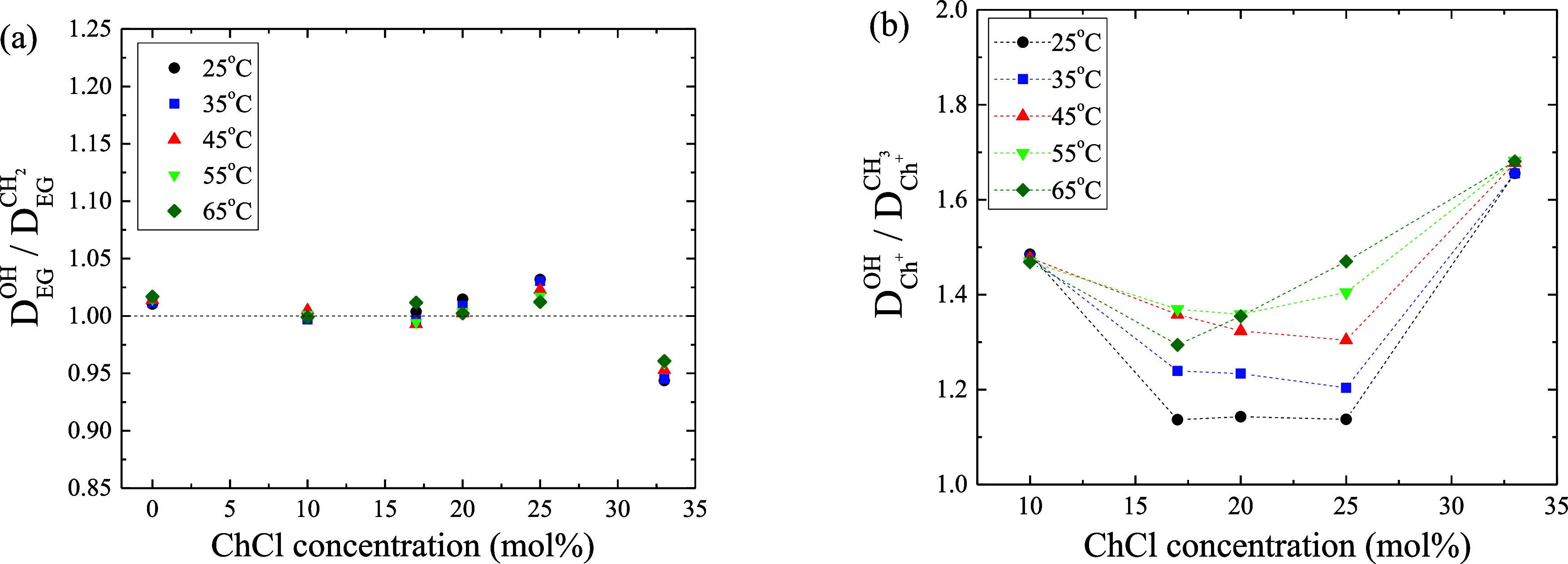
Ratio of (a) ${D_{\mathrm{EG}}}^{\mathrm{OH}}$ to ${D_{\mathrm{EG}}}^{{\mathrm{CH}}_{2}}$ and (b) ${D_{{\mathrm{Ch}}^{+}}}^{\mathrm{OH}}$ to ${D_{{\mathrm{Ch}}^{+}}}^{{\mathrm{CH}}_{3}}$ as a function of ChCl concentration, at different temperatures. Dashed lines are guides to the eye.

It is worth noting that while EG is also involved in the hydrogen exchange process, it does not have two *D* values. This is because in all samples, the number of EG molecules is greater than the number of choline molecules. As a result, all −OH groups of choline are involved in this exchange process with EG, but only a few −OH groups of EG take part in it. Specifically, only 6 to 25% of EG molecules can fully participate in this exchange process as the ChCl concentration increases from 10 to 33 mol %.

In the range of 17–25 mol % ChCl, which includes the eutectic concentration range, ${D_{{\mathrm{Ch}}^{+}}}^{\mathrm{OH}}$ / ${D_{{\mathrm{Ch}}^{+}}}^{{\mathrm{CH}}_{3}}$ shows a “flat minimum”, as depicted in Figure 4b. As the temperature increases, this minimum tends to become a “sharp minimum” and shifts toward lower concentrations. The presence of this minimum indicates a different hydrogen exchange process within the eutectic concentration range in comparison to outside this range in ChCl concentrations. This process appears to be less significant in this range and therefore the value of ${D_{{\mathrm{Ch}}^{+}}}^{\mathrm{OH}}$ tends to the value of ${D_{{\mathrm{Ch}}^{+}}}^{{\mathrm{CH}}_{3}}$ . An explanation for this result is presented in the following paragraph.

At compositions near the true eutectic (approximately 17–20 mol % ChCl), the hydrogen-bonding environment achieves an optimal configuration, as reported by the literature in a similar system.^40^ In this regime, the −OH group of the choline cation interacts strongly with EG and Cl^–^, creating a tightly bound local environment. This strong hydrogen-bonding network restricts the translational diffusion of the hydroxyl-associated protons relative to other parts of the choline molecule, notably the CH_3_ groups, which experience weaker interactions and are less hindered. Consequently, the hydrogen exchange process becomes less significant, and the diffusion measured for hydroxyl protons ( ${D_{{\mathrm{Ch}}^{+}}}^{\mathrm{OH}}$ ) tends to the value of the methyl-associated diffusion ( ${D_{{\mathrm{Ch}}^{+}}}^{{\mathrm{CH}}_{3}}$ ), where no such proton exchange occurs. Outside the eutectic concentration range, this highly structured hydrogen-bonding and proton-exchange network is either diluted (at lower ChCl concentrations) or overly disrupted (at higher concentrations), resulting in more difference between hydroxyl and methyl diffusion rates.

### 3.2 Dispersions of the Longitudinal Relaxation Rate by FFC-NMR

#### 3.2.1 Modeling of Relaxation Profiles

Fast field cycling (FFC) NMR relaxometry is a noninvasive low-field, low-resolution NMR technique that provides the dependence of the longitudinal relaxation time *T*_1_, or relaxation rate *R*_1_ = 1/*T*_1_, on the strength of the external magnetic field *B*_0_ (or Larmor frequency ω_0_ = γ*B*_0_, where γ is the nuclear gyromagnetic ratio) and this is called relaxation dispersion profile.^27–29,41^ It is important to note that this technique yields only a bulk, average T_1_ value, as broadband FFC lacks the chemical shift resolution needed to differentiate signals from given nuclei (e.g., ^1^H) in different molecular environments.

The molecular motion in liquids affects the interactions between molecules that possess nuclear spins. This movement is primarily a result of the rotation of the molecules and their relative translational diffusion, which are the principal reasons for the longitudinal magnetic relaxation of nuclei with spin *I* = 1/2, such as the hydrogen nucleus (^1^H).^32^ In this study, the longitudinal relaxation dispersion profiles of ^1^H in EG and Ch^+^ were modeled by considering translational diffusion and molecular rotations as the main relaxation mechanisms. Additionally, in samples containing EG-d6 or Ch-d9Cl, only the ^1^H–^1^H dipolar interactions were considered, while the heterodipolar coupling between hydrogen and deuterium was disregarded given its relatively small magnitude and rapid averaging.

The force-free-hard-sphere (FFHS) model^42,43^ was used to describe the effect of translational diffusion on intermolecular dipolar interactions. In spite of the fact that our samples consist of nonspherical molecules, the FFHS model has been employed successfully in similar systems, such as ionic liquids^44–46^ and glyceline.^23^ This model has a simple expression by assuming that the diffusion can be represented by finite jumps, in the Brownian limit *l* ^2^/6*d* ^2^ *→ 0*, where *l* ^2^ is the mean square jump distance defined by *l* ^2^ *≡* 6*D*τ, τ is the mean time between jumps and *D* is the self-diffusion coefficient. Then, the relaxation rate due to intermolecular interactions modulated in time by relative translational diffusion is given by

$$R_{1\mathrm{inter}\_\mathrm{Diff}}(\omega )=\frac{A_{D}}{dD_{12}}[\tilde{J}(z(\omega ))+4\tilde{J}(z(2\omega ))]$$ 1

where

$$A_{D}=\frac{8}{45}\pi \gamma^{4}\;\hbar^{2}{\left(\frac{\mu_{0}}{4\pi }\right)}^{2}n$$ 2

and

$$\tilde{J}(z)=\frac{1+\frac{5}{8}z+\frac{z^{2}}{8}}{1+z+\frac{z^{2}}{2}+\frac{z^{3}}{6}+\frac{z^{5}}{81}+\frac{z^{6}}{648}}$$ 3

*d* is the closest distance between two nuclei located in different molecules; *D*_12_ is the relative diffusion coefficient defined as the sum of the self-diffusion coefficients of the pair of molecules involved in the intermolecular interaction (*D*_12_ *= D*_1_ + *D*_2_); *n* is the number density of nuclei, ℏ is Plank’s constant divided by 2π and μ_0_ the vacuum magnetic permeability; ω = 2πν, where ν is the Larmor frequency, and $z\left(\omega \right)≡\sqrt{2\omega d^{2}/D_{12}}$ .

A Lorentzian function was used to describe the effect of molecular rotations on intramolecular dipolar interactions.^32,47^ Rotations were considered isotropic with an average correlation time and the corresponding relaxation rate is as follows:

$$R_{1\mathrm{intra}\_\mathrm{rot}}(\omega )=A_{R}\left[\frac{\tau_{R}}{1+\omega^{2}{\tau_{R}}^{2}}+\frac{4\tau_{R}}{1+(2\omega {)}^{2}{\tau_{R}}^{2}}\right]$$ 4

Here $A_{R}=\frac{3}{10}\gamma^{4}\hbar^{2}{\left(\frac{\mu_{0}}{4\pi }\right)}^{2}\frac{1}{r^{6}}$ is the amplitude reflecting the strength of the relevant ^1^H–^1^H dipolar interactions, *r* is the effective ^1^H–^1^H distance between two nuclei located in the same molecule and τ_R_ is the average rotational correlation time. τ_R_ is a characteristic time for rotations of the molecules as a whole. The shorter it is, the faster the molecular reorientations occur.

If it is assumed that the two processes described above are statistically independent and dominant in different time scales, the total ^1^H longitudinal relaxation rate for EG and Ch^+^ can be expressed as

$$R_{1\mathrm{total}}(\omega )=R_{1\mathrm{inter}\_\mathrm{Diff}}(\omega )+R_{1\mathrm{intra}\_\mathrm{rot}}(\omega )$$ 5

#### 3.2.2 Ethylene Glycol Dynamics

Figure 5a displays the ^1^H longitudinal relaxation rate dispersions of EG with 0, 10, 20, and 33 mol % Ch-d9Cl concentrations at −5 °C. The data indicate that the relaxation rate increases as the concentration of Ch-d9Cl increases. This is due to the higher viscosity and reduced molecular mobility of the solution at higher Ch-d9Cl concentrations. Figure 5b shows the ^1^H longitudinal relaxation rate dispersion of EG with 20 mol % Ch-d9Cl concentration and its fitting using the model of eq 5. A good agreement is observed between the experimental data and the model, within the experimental uncertainties. At lower frequencies, the ^1^H relaxation is dominated by translational diffusion, and while the diffusion contribution is frequency dependent, the rotational contribution is not. This suggests that rotations are faster than the time scales of our experiment, and hence their contribution to relaxation is constant. A comparable pattern was noted for pure EG and samples with 10 and 33 mol % Ch-d9Cl concentrations, as depicted in Figures S1, S2, and S3 in the Supporting Information (SI), respectively. In summary, the data analysis reveals clear low-frequency dispersion attributable to translational diffusion, whereas the flat high-frequency baseline reflects fast rotations, demonstrating that FFC-NMR can evaluate these two types of molecular motions that occur at different time scales.

**Figure 5:**
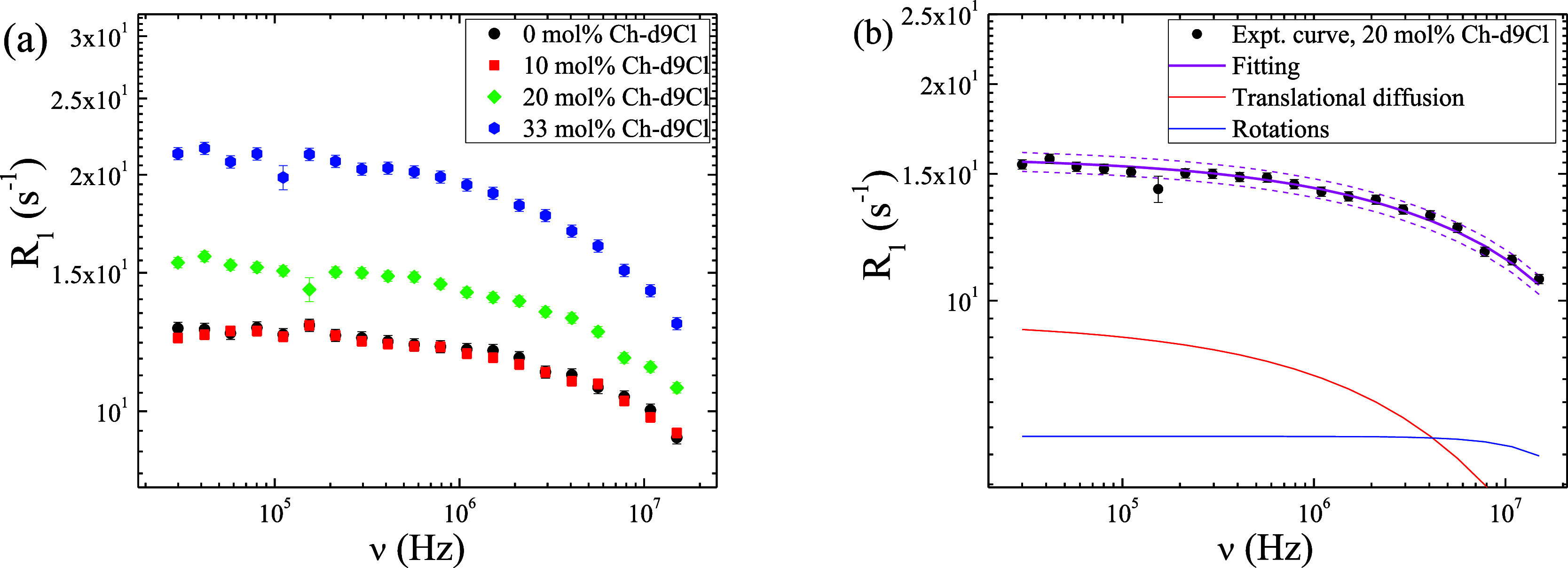
(a) ^1^H longitudinal relaxation rate dispersions of EG with 0, 10, 20, and 33 mol % Ch-d9Cl concentrations at −5 °C. (b) ^1^H longitudinal relaxation rate dispersion of EG with a 20 mol % Ch-d9Cl concentration, at −5 °C, and its corresponding fitting using the model given by eq 5. Translational (eq 1) and rotational (eq 4) contributions are additionally shown. Dashed lines represent the fitting error.

Even though Ch-d9Cl is not fully deuterated and part of the ^1^H signal in the relaxation experiments comes from Ch-d9, the measured signal and *R*_1_ values mainly belong to EG. Specifically, in the samples with 10, 20, and 33 mol % Ch-9Cl concentrations, 92%, 83%, and 71% of hydrogens belong to EG, respectively.

The parameters *d*, *A*_R_, and τ_R_ included in the model of eq 5 were estimated from the fittings, while the diffusion coefficients *D*_12_ *=* 2*D*_EG_ were fixed to the values obtained from our PFG-NMR diffusion measurements. The corresponding fitting parameters are presented in Table S1 in Supporting Information. Figure 6 shows the average rotational correlation times (τ_R_) of EG in samples that contain 0, 10, 20, and 33 mol % Ch-d9Cl, at −5 °C. As the Ch-9Cl concentration increases, this time becomes larger. These correlation times do not display any special feature across the eutectic concentration range (15–20 mol % ChCl) and only the viscosity effect is observed. This is consistent with our previous findings for diffusion coefficients (Figure 3).

**Figure 6:**
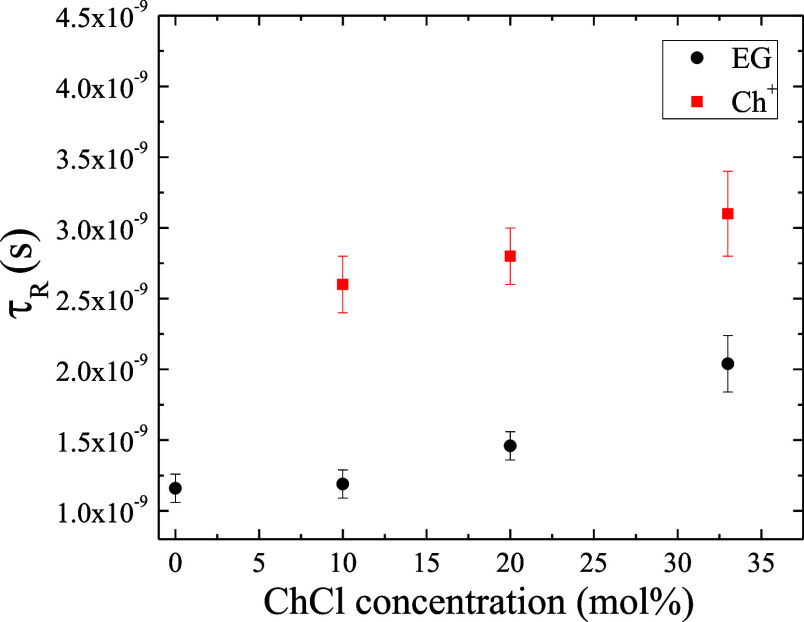
Average rotational correlation times of EG and Ch^+^ as a function of ChCl concentration, at −5 °C.

#### 3.2.3 Choline Dynamics

Figure 7a shows the ^1^H longitudinal relaxation rate dispersions of Ch^+^ for samples made of EG-d6 with varying ChCl concentrations, measured at −5 °C. The data suggest that the relaxation rate of Ch^+^ increases as the concentration of ChCl increases. This is attributed to the higher viscosity and reduced molecular mobility of the solution at higher ChCl concentrations. Figure 7b shows the ^1^H longitudinal relaxation rate dispersion of Ch^+^ in the sample made of EG-d6 with a 20 mol % ChCl concentration, along with its fitting using the model of eq 5. The model fits the experimental data quite well, within the experimental uncertainties. It is worth noting that, across the entire range of frequencies, the ^1^H relaxation is predominantly caused by molecular rotations. This pattern was also followed by the EG-d6 sample containing a 10 mol % ChCl concentration, as depicted in Figure S4 in the Supporting Information. In contrast, for the sample with a 33 mol % ChCl concentration, translational diffusion is the dominant relaxation mechanism across the entire range of frequencies, as shown in Figure S5. These results indicate that at low ChCl concentrations, choline cations are well-dispersed in the EG matrix, resulting in large intermolecular choline–choline distances. Consequently, intermolecular dipolar relaxation via translational diffusion is less significant, and intramolecular dipolar relaxation through molecular rotations becomes the primary mechanism. However, at 33 mol % ChCl, the increased choline concentration leads to closer intermolecular interactions, making translational diffusion the dominant relaxation pathway.

**Figure 7:**
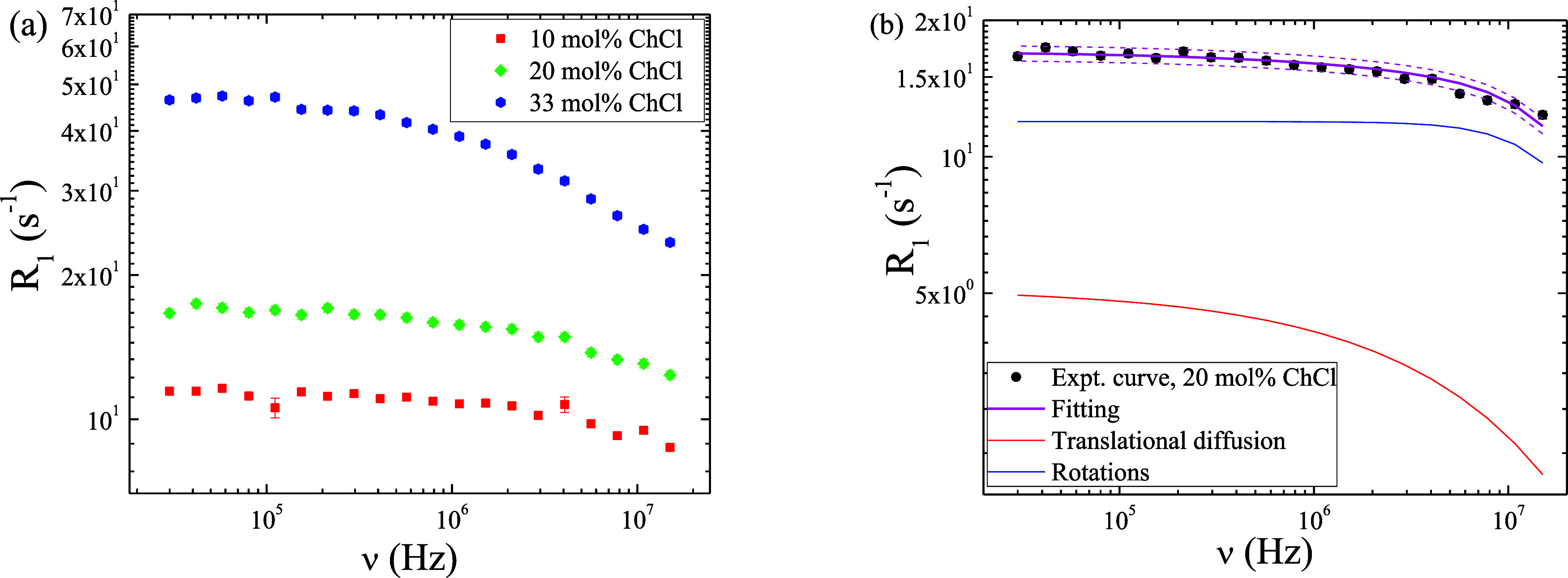
(a) ^1^H longitudinal relaxation rate dispersions of Ch^+^ in samples made of EG-d6 with 10, 20, and 33 mol % ChCl concentrations, at −5 °C. (b) ^1^H longitudinal relaxation rate dispersion of Ch^+^ in the sample that contains a 20 mol % ChCl concentration, at −5 °C, and its corresponding fitting using the model given by eq 5. Translational (eq 1) and rotational (eq 4) contributions are additionally shown. Dashed lines represent the fitting error.

The parameters *d*, *A*_R_, and τ_R_ included in the model of eq 5 were estimated from the fittings, while the diffusion coefficients *D*_12_ *=* 2*D*_Ch+_ were fixed to the values obtained from our PFG-NMR diffusion measurements. The corresponding fitting parameters are presented in Table S2 in the Supporting Information.

As a consequence of ^1^H exchange between the −OH group of choline and −OD groups of ethylene glycol, 7% of the total amount of ^1^H nuclei belong to ethylene glycol but they can be neglected as a first approach. Therefore, the measured signal for these samples was considered to predominantly arise from ^1^H in choline molecules.

The average rotational correlation times (τ_R_) of Ch^+^ in samples made of EG-d6 with 10, 20, and 33 mol % ChCl, at −5 °C, are displayed in Figure 6. As the ChCl concentration increases, this time becomes larger. These correlation times do not show any distinctive or anomalous behavior within the eutectic concentration range (15–20 mol % ChCl). Instead, their variation appears predominantly governed by the increase in bulk viscosity with higher ChCl content, in line with observations on translational diffusion behavior. This observation supports the hypothesis that rotational motion, as probed by τ_R_, is largely dictated by macroscopic viscosity rather than by localized structural or hydrogen-bonding reorganizations occurring at the eutectic.

### 3.3 Temperature-Dependent ^13^C Relaxation Experiments at Fixed Magnetic Field

To probe segmental rotational dynamics, we performed temperature-dependent ^13^C longitudinal relaxation (*T*_1_) measurements on ethylene glycol and choline carbons in EG–ChCl mixtures. Within the Bloembergen–Purcell–Pound (BPP) framework,^47^ the relaxation rate *R*_1_ = 1/*T*_1_ of each ^13^C nucleus is governed by fluctuations of the local ^13^C–^1^H dipolar couplings due to molecular reorientation:

$$\frac{1}{T_{1}}=N_{H}\frac{\mu_{0}^{2}}{4\pi }\frac{\hbar^{2}\gamma_{C}^{2}\gamma_{H}^{2}}{10r_{\mathrm{CH}}^{6}}\left[J\left(\omega_{H}-\omega_{C}\right)+3J\left(\omega_{C}\right)+6J\left(\omega_{H}+\omega_{C}\right)\right]$$ 6

where *N*_H_ is the number of attached protons, *r*_CH_ is the C–H distance, and *J*(ω) is the spectral density, modeled assuming isotropic rotational diffusion with correlation time τ_c_. This analysis yields an effective τ_c_ for each carbon site, which reflects the local friction experienced by that segment and can deviate from trends in the bulk viscosity.

We focus on ^13^C *T*_1_ rather than ^1^H *T*_1_ because carbon relaxation provides site-specific information on local friction that is often obscured in proton NMR. In these hydrogen-bonded mixtures, the ^1^H spins form a strongly coupled network subject to rapid spin diffusion and chemical exchange; consequently, ^1^H *T*_1_ is averaged over multiple environments and primarily reflects the global tumbling of the bulk liquid. By contrast, each ^13^C nucleus is isolated (weakly coupled to other carbons) and its relaxation is dominated by dipolar interactions with its directly bound protons. This provides a well-defined interaction geometry and cleanly resolved resonances, ensuring that the extracted τ_c_ values report on the rotational dynamics of individual segments rather than a bulk average.

It is important to distinguish the site-specific correlation time τ_c_ derived here from the rotational correlation time τ_R_ extracted from FFC relaxometry. In the FFC analysis, the dispersion profiles were modeled using the FFHS framework, where τ_R_ represents an average tumbling time for the entire choline or EG molecule, common to all protons on that moiety. In contrast, the ^13^C-derived τ_c_ describes local segmental reorientation of specific C–H vectors (EG CH_2_, choline CH_2_–N_α_, CH_2_–N_β_, and NMe). While τ_R_ and τ_c_ probe different motional modes (whole-molecule tumbling versus local segmental rotation) and distinct frequency windows, they reveal consistent qualitative trends: both parameters indicate a slowing of choline dynamics with increasing ChCl content and a comparatively weak sensitivity of EG dynamics up to ∼20 mol %. This convergence supports a consistent physical picture of viscosity-dominated dynamics modulated by subtle, site-dependent interactions near the eutectic composition.

#### 3.3.1 Temperature Dependence and Accessible Motional Regime

Figure 8a–h show the ^13^C *T*_1_ data as a function of inverse temperature for the four compositions studied as well as neat EG. All sites display the expected BPP-like behavior, with *T*_1_ decreasing upon cooling, flattening at low temperature, and then tending toward a minimum that lies at or below the cold end of our experimental window for most salt-containing mixtures. Within the accessible −45 to 35 °C range, the *T*_1_(T) curves for EG–ChCl mixtures generally show monotonically decreasing *T*_1_ upon cooling with incipient flattening at the lowest temperatures, and well-resolved minima are not observed for most sites. This indicates that the system is on or approaching the fast-motion side of the BPP dispersion for many sites, although some compositions at high ChCl content may lie in a transition regime rather than deep in the extreme-narrowing limit. In the absence of fully developed minima, we adopt the standard fast-motion approximation 1/*T*_1_ ∝ τ_*c*_ as a first-order model and use *T*_1_ (25 °C) together with the Solomon–Bloembergen relation to extract effective τ_c_ values.^31,32^ These values should be interpreted as comparative measures of local reorientational friction across composition and site, rather than as precise absolute correlation times. This approach contrasts with our previous glyceline study,^23^ where well-developed ^13^C *T*_1_ minima for each carbon enabled a direct mapping between the position of the minima and τ_c_(T) over a broad

**Figure 8:**
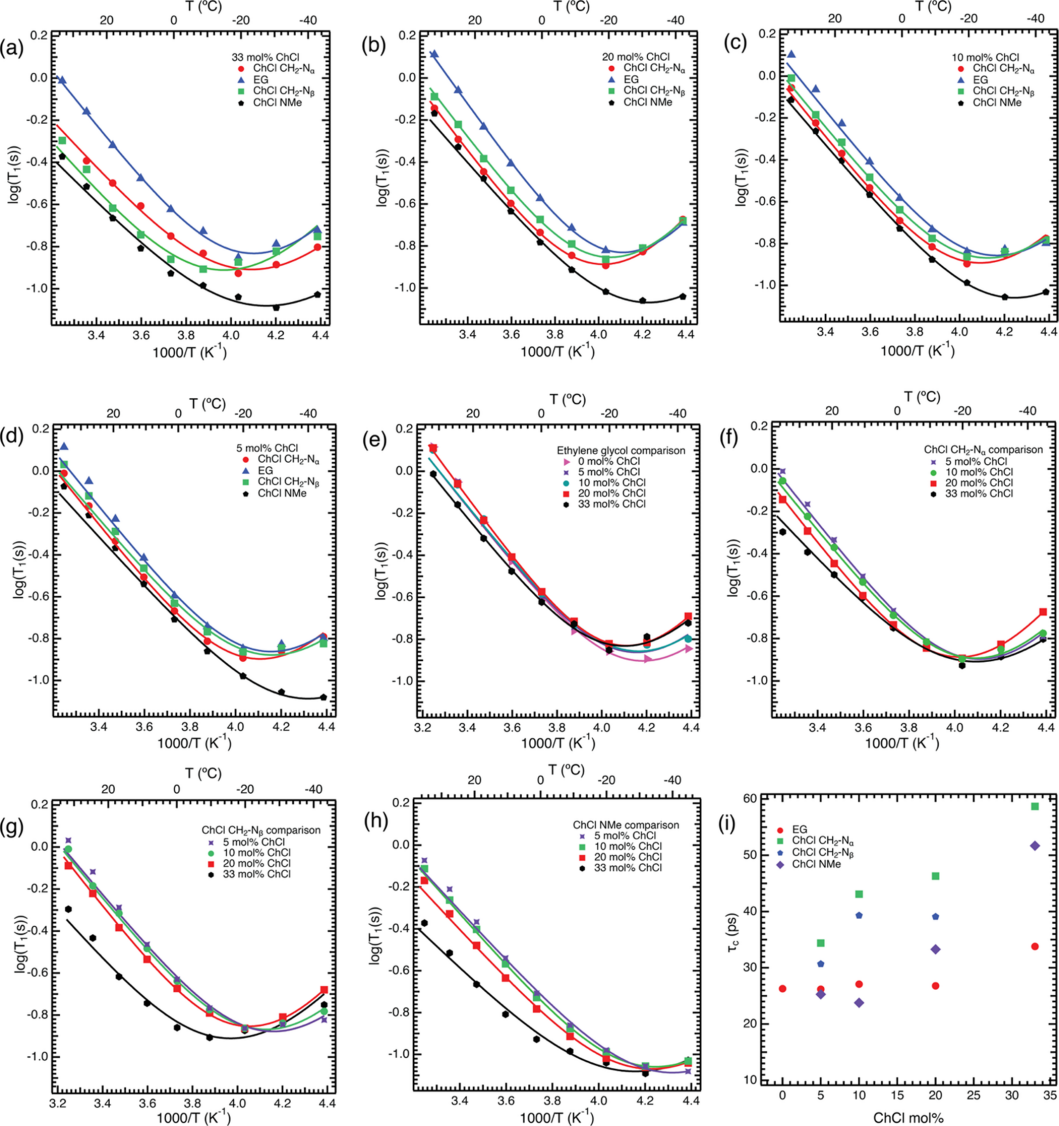
(a–d) ^13^C NMR relaxation data of the ChCl-EG system as a function of composition. Here ChCl CH_2_–N_α_ is the carbon alpha to the ChCl ammonium, ChCl CH_2_–N_β_ is the carbon beta to the ChCl ammonium, and ChCl NMe is the methyl groups on the ammonium. (e–h) ^13^C NMR relaxation data comparing the carbon types as a function of composition. (i) Composition dependence of τ_c_ extracted from ^13^C *T*_1_ fits at 25 °C.

range; in the present EG–ChCl system, the ^13^C data chiefly constrain the relative amplitude of τ_c_ at 25 °C as a function of composition and site. We note that modest deviations from the extreme-narrowing assumption (e.g., ωτ_*c*_ ∼ 0.3 – 0.5 instead of ≪1) would change the absolute τ_c_ values by factors of order unity but would not alter the qualitative trends with composition that form the basis of our discussion.

#### 3.3.2 Composition Dependence and Site-Resolved Trends

The correlation times extracted at 25 °C (Figure 8i) reveal a clear hierarchy of local dynamics that is qualitatively consistent with chemical intuition. The choline *N*-methyl carbons exhibit the shortest τ_*c*_ at all compositions, reflecting fast internal methyl rotation and their peripheral, relatively weakly hydrogen-bonded environment. The CH_2_–N_α_ and CH_2_–N_β_ carbons, which reside closer to the quaternary ammonium center and the hydroxyl group, reorient more slowly and show systematically longer τ_c_ values. The EG methylene carbon reorients on a similar time scale to the choline methylenes, with only modest variation up to the near-eutectic composition.

As the ChCl mole fraction increases from 5 to 33 mol %, the ^13^C *T*_1_ at 25 °C decreases for all choline sites, consistent with larger 1/*T*_1_ and hence longer τ_c_ in the fast-motion limit. This trend indicates that choline segmental rotation progressively slows as the liquid becomes more ionic and viscous, in line with the substantial viscosity increase reported from independent densimetry and rheology studies. The CH_2_–N_α_ carbon, in particular, shows a steady growth in τ_c_ with salt content and a hint of enhanced hindrance (a shoulder or weak maximum) near 17–20 mol % ChCl, within the experimental uncertainty. By contrast, the *N*-methyl τ_c_ exhibits the most complex composition dependence: it decreases slightly from 5 to 10 mol % (−6%, within experimental uncertainty), then increases progressively at 20 mol % and more markedly at 33 mol %, highlighting the peripheral nature of this site and its initially weak but gradually strengthening coupling to the hydrogen-bond network at higher salt concentrations.

The EG methylene correlation time exhibits a different pattern: τ_c_ remains nearly constant from 0 up to 20 mol % ChCl (varying by less than 3%) and then increases by approximately 25% at 33 mol %. This “plateau-then-rise” behavior suggests that, up to the near-eutectic composition, the local friction experienced by EG segments is relatively robust against moderate salt addition, even though the macroscopic viscosity already grows significantly over this range. Only in the most concentrated mixture does the local environment of EG change enough to produce a clear slowing of its reorientation.

Altogether, the ^13^C results indicate that most segmental correlation times either increase monotonically or remain nearly constant with increasing ChCl content; they do not exhibit a pronounced global minimum at the eutectic composition. The notable feature of the near-eutectic region is instead a subtle site-specific deviation: the CH_2_–N_α_ site appears slightly more hindered around 17–20 mol % than would be expected from a simple extrapolation of the low- and high-salt trends, whereas the other carbons show smoother composition dependence.

#### 3.3.3 Connection to Diffusion and the Near-Eutectic Regime

The site-resolved ^13^C trends also agree with the PFG-NMR diffusion measurements. The self-diffusion coefficients of EG and choline decrease smoothly with ChCl content over the 0–33 mol % range, broadly tracking the viscosity, with no sharp anomaly at the near-eutectic composition in the individual *D* values. However, the ratio of the diffusion coefficients inferred from the choline OH and methyl proton environments shows a shallow minimum near 17–20 mol % ChCl, indicating that the hydroxyl-bearing “head” of choline experiences slightly stronger translational drag from the hydrogen-bond network at these compositions than the more hydrophobic methyl “tail.” This dynamical asymmetry is mirrored in the ^13^C data: CH_2_–N_α_, which is closest to the charged ammonium headgroup, displays the clearest hint of extra hindrance in τ_c_ near the near-eutectic composition, whereas NMe and EG show more gradual trends.^13,33,48^

Taken together with previous conductivity data and hole-theory analyses, which place a broad conductivity maximum and a transport crossover near *x*_ChCl_ ≈ 0.2, these observations suggest that the near-eutectic region is best viewed as a subtle dynamical crossover rather than a singular structural state. In this composition range, the EG-rich hydrogen-bond network is still sufficiently cohesive to exert maximal friction on the charged headgroup region of choline, while the surrounding microstructure begins to reorganize into more ionic, heterogeneous domains. Beyond this point, at 33 mol % ChCl, the liquid becomes more salt-rich and ionic-liquid-like; segmental τ_c_ for all sites increase, but the relative differences between EG and choline sites diminish as the network fragments into smaller ionic clusters.

In summary, the ^13^C *T*_1_ analysis shows that rotational dynamics in EG–ChCl mixtures are largely viscosity-dominated and compositionally smooth, with only modest, site-specific deviations near the thermodynamic eutectic. The near-eutectic composition does not generate a universal anomaly in rotational correlation times; instead, it marks a regime where the hydrogen-bond/ionic network exerts slightly enhanced friction on specific charged segments of choline while most other dynamical observables follow the monotonic increase in viscosity. This nuanced picture aligns with the PFG and FFC results and reinforces the view that DES dynamics are governed by a balance between bulk viscosity and local microenvironment, rather than by a single special composition.

### 3.4 2D NOESY: Probing Local Structure and Exchange in EG–ChCl Mixtures

Nuclear Overhauser Effect Spectroscopy (NOESY) is a two-dimensional NMR technique that reports on through-space dipolar couplings between protons that are typically within ∼5 Å of one another. In contrast to scalar-coupling based methods such as COSY, which map through-bond connectivity, NOESY cross-peaks arise from cross-relaxation between nearby spins and therefore encode short-range spatial proximity and the time scales of molecular motion that modulate dipolar interactions. By following the buildup and decay of cross-peak intensities as a function of mixing time, one can distinguish direct dipolar contacts from longer-range spin diffusion and identify contributions from chemical exchange processes, such as proton transfer between hydrogen-bond donors and acceptors.

These capabilities are particularly valuable for DESs, where structure and dynamics are governed by a dense, heterogeneous hydrogen-bond network. Most of what is known about DES microstructure has come from scattering experiments, dielectric spectroscopy, and molecular dynamics simulations, which collectively point to nanoscale heterogeneity and the coexistence of ionic and HBD-rich domains in choline chloride–based DESs.^7,12,14,15,24,33^ NOESY complements these methods by providing a direct, site-resolved picture of which molecular fragments are in close spatial proximity and how long those contacts persist on the NMR time scale.

In the present EG–ChCl system, NOESY offers a way to connect the composition-dependent dynamics inferred from diffusion, FFC relaxometry, and ^13^C *T*_1_ measurements to an explicit pattern of local contacts. By recording series of NOESY spectra over mixing times from 50 to 800 ms for compositions spanning 0–33 mol % ChCl, we can (i) map intra- and intermolecular contacts between choline headgroup, methyl, and ethylene glycol protons; (ii) assess how the density and strength of short-range interactions evolve from EG-rich to salt-rich regimes; and (iii) search for signatures of slow hydroxyl exchange or clustering that might be enhanced near the near-eutectic composition. In this way, NOESY serves as both a structural and dynamic probe, helping to determine whether the eutectic regime is distinguished primarily by its hydrogen-bond topology, by exchange kinetics, or by a combination of both.

To complement the translational and rotational dynamics obtained from PFG diffusion, FFC relaxometry, and ^13^C *T*_1_ measurements, we acquired a series of 2D ^1^H NOESY spectra on EG–ChCl mixtures at 5, 10, 20, and 33 mol % ChCl. For each composition, spectra were recorded at 25 °C with mixing times of 50, 200, 400, and 800 ms. The spectra were reprocessed with consistent apodization, phasing, and frequency windows. Because these DES mixtures are highly viscous and feature extensive hydrogen bonding and exchange, spin diffusion and OH exchange become significant at times greater than or equal to 200 ms. We therefore treat the NOESY spectra as qualitative probes of relative contact patterns rather than as a basis for quantitative distance extraction.^49^

Figure 9 shows the NOESY series for the 20 mol % ChCl mixture, which lies close to the true eutectic composition. At the shortest mixing time (50 ms), the spectrum is dominated by diagonal peaks from ethylene glycol and choline, with a small number of well-defined cross-peaks linking choline headgroup resonances (CH_2_–N_α_, CH_2_–N_β_) to the EG CH_2_/OH region. These early time cross-peaks signal frequent, short-range encounters between the choline headgroup and the polyol backbone within the hydrogen-bonded network.

**Figure 9:**
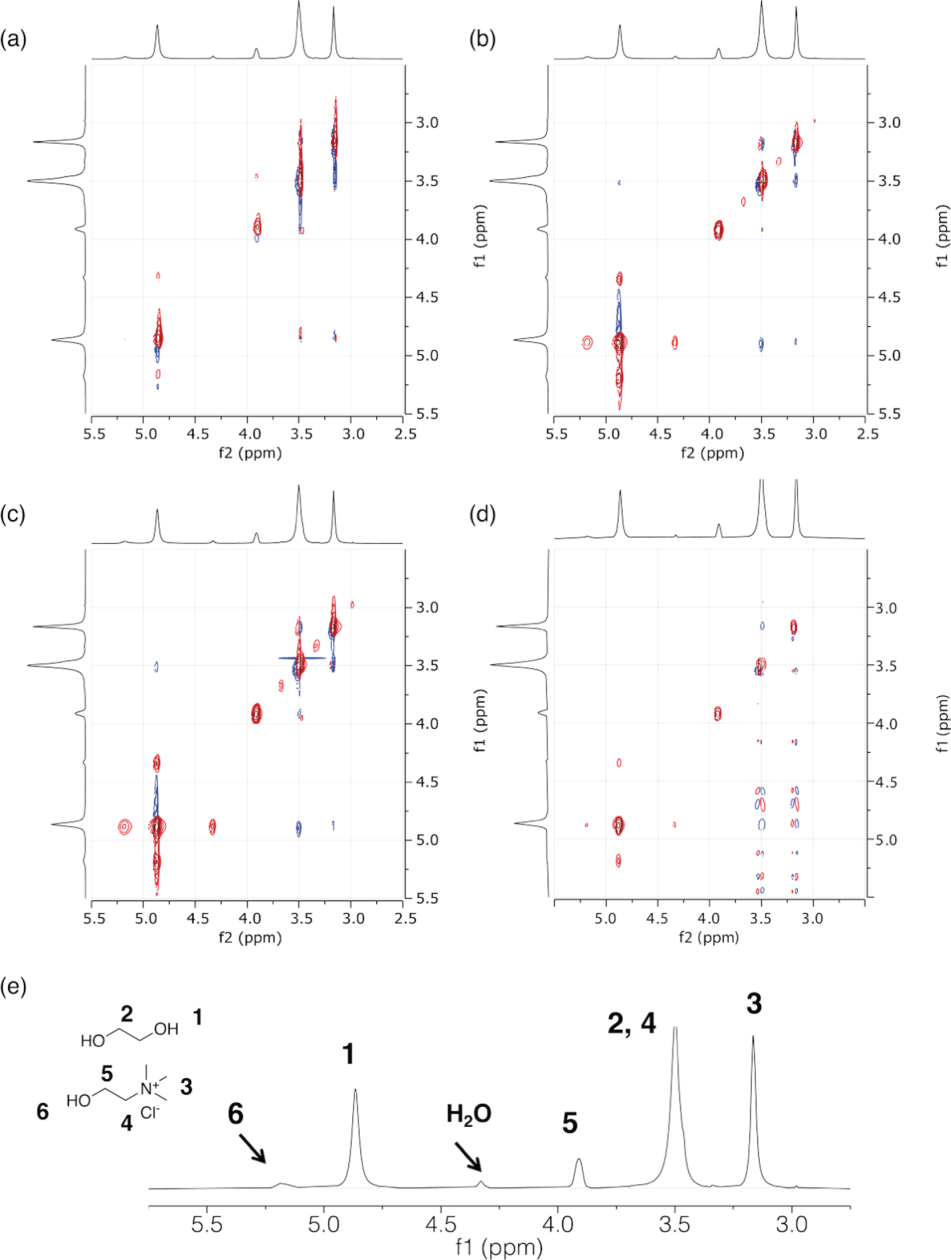
2D NOESY spectra for 20 mol % ChCl in EG at 25 °C for four different mixing times (a) 50 ms, (b) 200 ms, (c) 400 ms, and (d) 800 ms (threshold lessened to reduce *t*_1_ noise). (e) 1–D ^1^H NMR spectrum of 20 mol % ChCl in EG.

As the mixing time is increased to 200 and 400 ms, the number and intensity of cross-peaks grow substantially. Choline–choline and choline–EG cross-peaks form an interconnected web linking the choline *N*-methyl, CH_2_–N_α_, and CH_2_–N_β_ resonances to multiple EG environments. This dense pattern of cross-peaks indicates a high local coordination density in which choline cations and EG molecules share a common hydrogen-bonded environment and undergo rapid reconfiguration on the NOESY time scale. Cross-peaks involving OH-bearing sites maintain the same sign as the diagonal at long mixing times, consistent with a significant contribution from chemical exchange between hydrogen-bonded OH sites superimposed on the dipolar NOE. The coexistence of strong EG–choline NOEs and OH-exchange–driven cross-relaxation at 20 mol % ChCl is therefore consistent with a highly interconnected, dynamically reconfiguring hydrogen-bond network.

At the longest mixing time (800 ms), the 20 mol % spectrum becomes heavily cross-peaked and partially congested, reflecting the combined effects of spin diffusion, exchange, and relaxation losses in this viscous medium as well as demonstrating pronounced *t*_1_ noise. Nevertheless, the overall pattern remains consistent with an EG–ChCl liquid in which choline cations are intimately mixed with EG and chloride, rather than phase-separated or weakly solvated. This picture aligns with our diffusion and ^13^C relaxation data, which converge on a consistent but site-resolved picture: PFG diffusion highlights enhanced frictional drag at the hydroxyl-bearing end of choline near the eutectic, while the site-resolved ^13^C τ_c_ reveals analogous enhanced hindrance at CH_2_–N_α_, adjacent to the charged ammonium group. These observations indicate that both the hydrogen-bonding and ionic functionalities of the choline cation experience strengthened coupling to the EG/Cl^–^ network near the eutectic, manifested at distinct molecular sites.

Additional NOESY spectra for 5, 10, and 33 mol % ChCl are presented in Figures S6–S8. Briefly, at 5 mol % ChCl the spectra are still dominated by EG, and only weak choline–EG cross-peaks appear even at long mixing times, consistent with a largely EG-rich network in which the ionic component is sparsely distributed. At 10 mol %, EG–choline cross-peaks become more prominent and choline–choline contacts emerge, indicating more frequent encounters as ion-rich domains start to form. At 33 mol %, the spectra remain strongly cross-peaked but become more congested and somewhat broader, suggesting a transition to a salt-rich, microheterogeneous regime in which EG experiences a different local environment and distinct EG–choline contacts are partially subsumed into a more disordered ionic-liquid-like matrix.

Taken together, the NOESY results support the view that the near-eutectic composition marks a crossover in local organization rather than a unique structure. As ChCl is added from 5 to 20 mol %, NOESY reveals a clear increase in the density and connectivity of EG–choline contacts and in OH exchange pathways, in line with the slowdown of the choline OH-bearing end seen in the diffusion ratio ${D_{{\mathrm{Ch}}^{+}}}^{\mathrm{OH}}$ / ${D_{{\mathrm{Ch}}^{+}}}^{{\mathrm{CH}}_{3}}$ and the site-specific trends in ^13^C-derived correlation times. Beyond 20 mol %, the NOESY spectra show that the system remains highly interactive but evolves toward a more strongly salt-rich, microheterogeneous structure, consistent with the transport crossover inferred from conductivity and hole-theory analyses.

### 3.5 Integrated NMR Perspective on Dynamics in EG–ChCl

The combination of PFG diffusion, FFC relaxometry, ^13^C *T*_1_ measurements, and NOESY provides a consistent, multiscale picture of dynamics in EG–ChCl and clarifies the characteristic behavior of the near-eutectic composition.

PFG-NMR shows that, across 0–33 mol % ChCl, both ethylene glycol and the choline cation slow down steadily as salt is added, broadly tracking the strong, monotonic increase in bulk viscosity reported in the literature. Ethylene glycol diffuses faster than choline at all compositions, and neither species exhibits a sharp anomaly at the true eutectic. The only composition-sensitive feature is a shallow minimum in the ratio ${D_{{\mathrm{Ch}}^{+}}}^{\mathrm{OH}}$ / ${D_{{\mathrm{Ch}}^{+}}}^{{\mathrm{CH}}_{3}}$ near 17–20 mol % ChCl, indicating that the hydroxyl-bearing end of choline experiences slightly stronger frictional drag from the hydrogen-bond network in this regime than the methyl terminus. This behavior is subtle, but it already hints that the headgroup and tail of the cation do not “see” the same local environment.

Fast-field-cycling ^1^H relaxometry complements these translational measurements by reporting on averaged rotational and translational motions on nanosecond to microsecond time scales. The field-dependent *R*_1_(ω) dispersions are well captured, to first order, by a force-free hard-sphere–type description in which correlation times grow with increasing viscosity. In other words, from the point of view of the proton bath, the liquid behaves as a highly viscous but compositionally smooth medium: adding ChCl primarily slows overall reorientation and translational diffusion without introducing a dramatic new relaxation mechanism at the eutectic. At the same time, the quality of the hard-sphere fits and the need for multiple motional modes underscore that the system is not a perfectly homogeneous continuum and that microheterogeneity is present.

The site-resolved ^13^C *T*_1_ measurements sharpen this picture by isolating local rotational friction for specific carbon sites. When the data are analyzed within the standard dipolar relaxation framework, the extracted correlation times at 25 °C reveal three robust trends. First, the choline *N*-methyl carbons retain the shortest τ_c_ at all compositions, reflecting fast internal rotation and relatively weak engagement with the hydrogen-bond network. Second, the ethylene glycol methylene carbon shows a composition-independent “plateau” up to roughly 20 mol % ChCl, followed by a modest speeding-up at 33 mol %, suggesting that the local friction experienced by EG is remarkably insensitive to salt addition until the liquid becomes strongly salt-rich. Third, the choline CH_2_–N_α_ site exhibits slightly enhanced hindrance near 17–20 mol % ChCl relative to its otherwise monotonic trend, echoing the shallow minimum in ${D_{{\mathrm{Ch}}^{+}}}^{\mathrm{OH}}$ / ${D_{{\mathrm{Ch}}^{+}}}^{{\mathrm{CH}}_{3}}$ . Taken together, these observations indicate a partial decoupling between macroscopic viscosity and local reorientational dynamics: as the extended EG network is disrupted into smaller, ion-coordinated domains, certain molecular segments (NMe, EG backbone) can reorient more freely, while others (the OH-bearing headgroup region) remain strongly coupled to the evolving hydrogen-bond network.

The NOESY experiments then provide a qualitative structural fingerprint of the short-range contacts and exchange pathways that underlie these dynamical trends. At low salt content (5 mol % ChCl, Figure S6), the spectra are still dominated by intramolecular EG cross-peaks, and choline–EG contacts remain sparse even at long mixing times, consistent with a largely EG-rich network. At intermediate composition (10 mol %, Figure S7), EG–choline cross-peaks become more numerous, indicating more frequent short-range encounters as ion-rich regions begin to develop. Near the eutectic (20 mol % ChCl, Figure 9), the NOESY spectra show the richest pattern of choline–EG cross-peaks within the series, consistent with a high density of short-range encounters between choline headgroup and EG protons on the NOESY time scale. We emphasize that, without detailed buildup curves, these spectra cannot unambiguously disentangle direct dipolar NOE from contributions due to spin diffusion; our interpretation is therefore restricted to the relative evolution of cross-peak patterns with composition and mixing time. At 33 mol % ChCl (Figure S8), cross-peaks remain abundant but become more congested and broadened, pointing to a salt-rich, microheterogeneous regime in which EG experiences a more disordered, ionic-liquid-like environment. For a subset of prominent cross-peaks (for example, choline *N*-methyl to EG CH_2_), the initial increase in intensity between 50 and 200 ms is approximately linear, suggesting that these contacts contribute directly to the NOE at short mixing times; at longer times, spin diffusion and exchange are expected to dominate.

Viewed together, the four NMR approaches converge on a coherent picture. PFG and FFC show that average translational and rotational motions are largely viscosity-dominated and do not display a dramatic anomaly at the eutectic composition. Site-resolved ^13^C *T*_1_ and NOESY, however, reveal that local friction, headgroup–tail decoupling, and short-range EG–choline contacts evolve nontrivially with composition, with the near-eutectic region marking a crossover where the hydrogen-bond network is most effective at coupling the hydroxyl-bearing choline headgroup into the EG/Cl^–^ matrix.

## 4 Conclusions

We have used pulsed-field-gradient diffusion, fast-field-cycling relaxometry, site-resolved ^13^C *T*_1_ measurements, and NOESY to develop an integrated, molecular-scale picture of dynamics in ethylene glycol–choline chloride mixtures over 0–33 mol % ChCl. Viewed against recent thermodynamic and transport work, our results show that, at the coarsest level, EG–ChCl behaves as a viscous but compositionally smooth liquid: both translational diffusion and average rotational motions slow down monotonically with increasing ChCl content, broadly tracking the strong rise in bulk viscosity, and we do not observe a sharp, universal dynamical anomaly at the eutectic composition near 17–20 mol % ChCl.

On top of this viscosity-dominated background, more subtle, site- and probe-dependent composition effects emerge. The diffusion ratio ${D_{{\mathrm{Ch}}^{+}}}^{\mathrm{OH}}$ / ${D_{{\mathrm{Ch}}^{+}}}^{{\mathrm{CH}}_{3}}$ shows a shallow depression near the eutectic within experimental uncertainty, the ^13^C-derived correlation time for the CH_2_–N_α_ site exhibits a small deviation from an otherwise monotonic trend, and NOESY spectra in the same composition range display a richer pattern of choline–EG cross-peaks and OH-involving correlations. We interpret these features as modest signatures of changes in local hydrogen-bond topology and headgroup–tail coupling, rather than as evidence for a singular, structurally exceptional eutectic state. In this view, the eutectic region marks a crossover in local organization and transport mechanism, between solvent-rich and salt-rich regimes, rather than a point where all dynamical properties reach an extremum.

Taken together, the four NMR approaches support a hierarchical view of dynamics in EG–ChCl. Bulk transport quantities such as viscosity and average diffusion provide a largely continuous, composition-dependent baseline, while local friction, segment-resolved mobility, and short-range EG–choline contacts show more nuanced and composition-sensitive behavior that is most pronounced near the eutectic region. This multitechnique NMR framework illustrates how global and local contributions to dynamics in deep eutectic solvents can be disentangled, and it underscores the importance of considering both macroscopic transport and site-specific mobility when selecting and tuning DES compositions for electrochemical, separation, and catalytic applications.

## Supplementary Material



## Notes

Supplementary figures for longitudinal relaxation rate profiles of choline and ethylene glycol with their corresponding fittings and values of fitting parameters; sensitivity analysis of rotational correlation time estimates from the experimental ^13^C *T*_1_ data; and 2D NOESY spectra of ethylene glycol with 5, 10, and 33 mol % ChCl (PDF)

## §

IFEG – CONICET and National University of Cordoba, Cordoba, Argentina
