# Supplementary material for: Insights into Rotational and Translational Dynamics in Mixtures of Ethylene Glycol and Choline Chloride Using Nuclear Magnetic Resonance Techniques
Source: J Phys Chem B. 2026 May 28;130(23):5939–52. doi: 10.1021/acs.jpcb.6c01832 (PMC13267078; doi:10.1021/acs.jpcb.6c01832)
Supplement: Supplementary file 1 [file jp6c01832_si_001.pdf]

# Supporting Information

# Insights into Rotational and Translational Dynamics in mixtures of ethylene glycol and choline chloride using Nuclear Magnetic Resonance techniques

Carla C. Fraenza[a,*,1], Ramez A. Elgammal[b], Thomas A. Zawodzinski[b], Steven G. Greenbaum[a,*]

[a]Department of Physics and Astronomy, Hunter College of CUNY, New York, New York 10065, United States.
[b]Department of Chemical and Biomolecular Engineering, University of Tennessee-Knoxville, Knoxville, Tennessee 37996, United States.

*Corresponding authors
E-mail: carla.cecilia.fraenza@unc.edu.ar (Carla C. Fraenza), sgreenba@hunter.cuny.edu (Steven G. Greenbaum).
[1]Present address: IFEG – CONICET and National University of Cordoba, Cordoba, Argentina.

**Dispersions of the Longitudinal Relaxation Rate: Ethylene Glycol Dynamics**

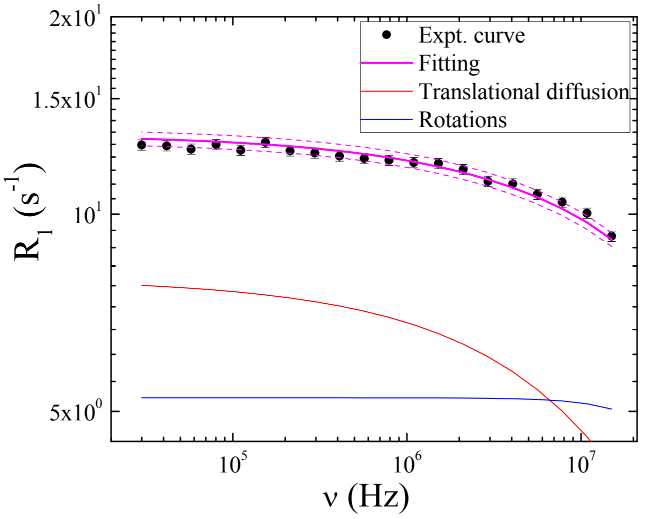


**Figure S1.** $^{1}$H longitudinal relaxation rate dispersion of pure EG at -5 °C and its fitting using the model given by Equation 5 of main manuscript. Translational (Equation 1) and rotational (Equation 4) contributions are additionally shown. Dashed lines represent the fitting error.

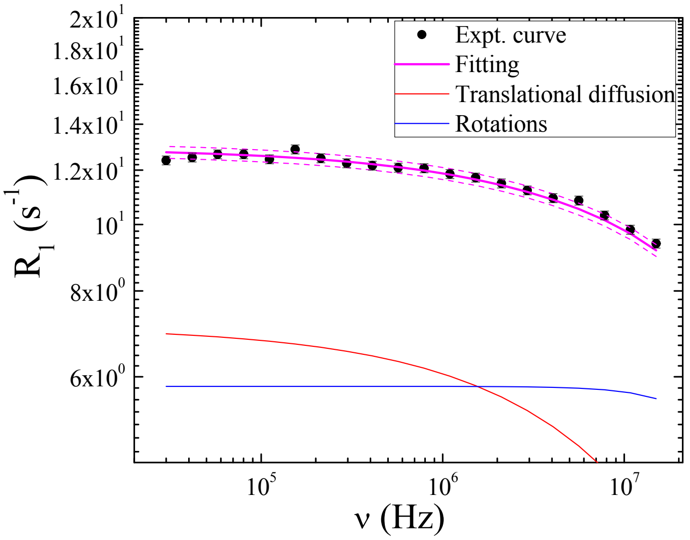


**Figure S2.** $^{1}$H longitudinal relaxation rate dispersion of EG with a 10 mol% Ch-d9Cl concentration, at -5 °C, and its fitting using the model given by Equation 5 of main manuscript. Translational (Equation 1) and rotational (Equation 4) contributions are additionally shown. Dashed lines represent the fitting error.

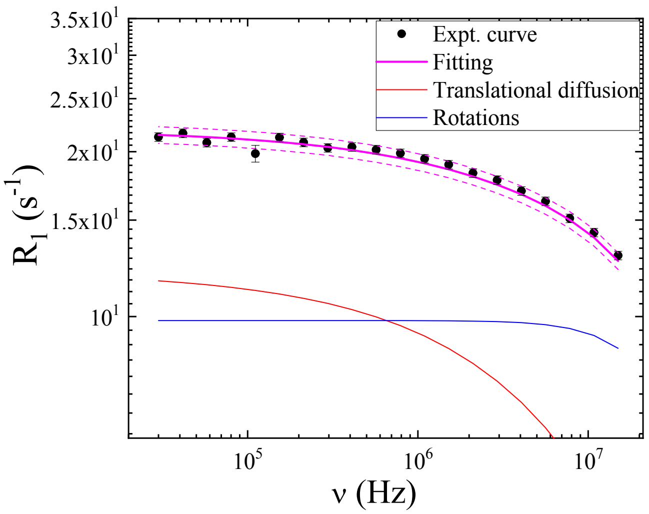


**Figure S3.** [1]H longitudinal relaxation rate dispersion of EG with a 33 mol% Ch-d9Cl concentration, at -5 °C, and its fitting using the model given by Equation 5 of main manuscript. Translational (Equation 1) and rotational (Equation 4) contributions are additionally shown. Dashed lines represent the fitting error.

**Table S1.** Fitting parameters for the [1]H relaxation rate profiles of ethylene glycol in samples that contain different Ch-d9Cl concentrations, at -5°C.

| **ETHYLENE GLYCOL DYNAMICS** | | | | |
|---|---|---|---|---|
| **Parameters** | **Ch-d9Cl concentration (mol%)** | | | |
| | 0 | 10 | 20 | 33 |
| $\boldsymbol{d}$ **[m] × 10**$^{-10}$ | 4.5±0.3 | 4.5±0.2 | 4.7±0.3 | 4.7±0.3 |
| $D_{EG} = D_{12}/2$ **[m²/s] × 10**$^{-12}$ | 14.5±0.3 | 14.0±0.2 | 10.1±0.1 | 6.0±0.1 |
| $A_R$ **[1/s²] × 10**$^{8}$ | 9.1±0.6 | 9.8±0.4 | 8.8±0.3 | 9.7±0.3 |
| $\tau_R$ **[s] × 10**$^{-9}$ | 1.2±0.1 | 1.2±0.1 | 1.5±0.1 | 2.0±0.2 |

**Dispersions of the Longitudinal Relaxation Rate: Choline Dynamics**

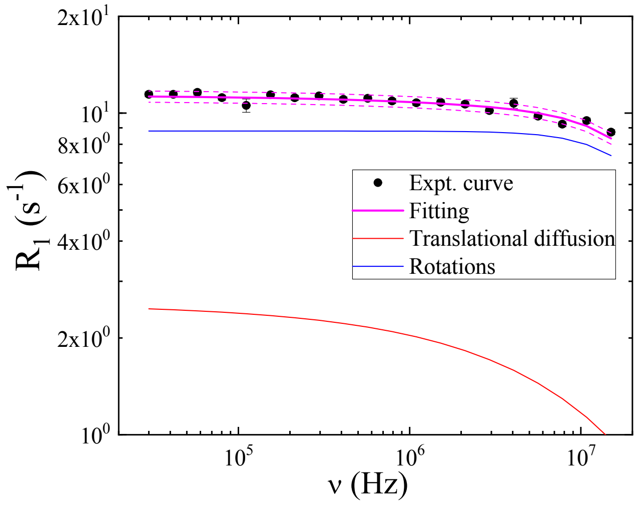


**Figure S4.** [1]H longitudinal relaxation rate dispersion of $Ch^+$ in the sample made of EG-d6 with a 10 mol% ChCl concentration, at -5 °C, and its corresponding fitting using the model given by Equation 5. Translational (Equation 1) and rotational (Equation 4) contributions are additionally shown. Dashed lines represent the fitting error.

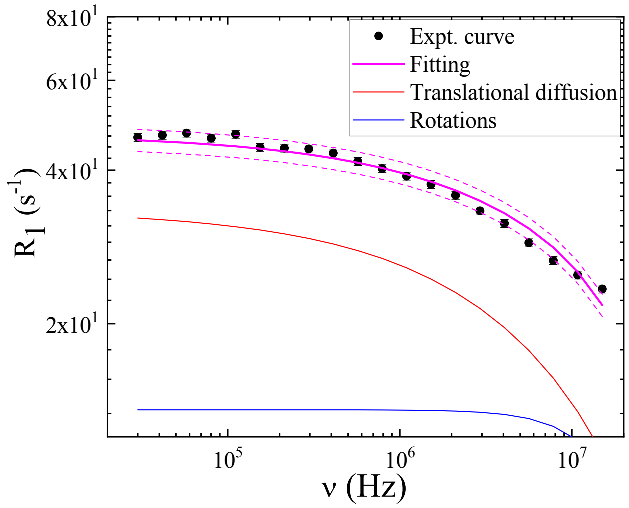


**Figure S5.** [1]H longitudinal relaxation rate dispersion of $Ch^+$ in the sample made of EG-d6 with a 33 mol% ChCl concentration, at -5 °C, and its corresponding fitting using the model given by Equation 5. Translational (Equation 1) and rotational (Equation 4) contributions are additionally shown. Dashed lines represent the fitting error.

**Table S2.** Fitting parameters for the $^1$H relaxation rate profiles of $Ch^+$ in samples that contain different ChCl concentrations, at -5°C.

| CHOLINE DYNAMICS | | | |
|---|---|---|---|
| **Parameters** | **ChCl concentration (mol%)** | | |
| | 10 | 20 | 33 |
| $\boldsymbol{d}$ **[m] × 10$^{-10}$** | 4.8±0.2 | 4.5±0.3 | 3.0±0.1 |
| $\boldsymbol{D_{Ch+} = D_{12}/2}$ **[m$^2$/s] × 10$^{-12}$** | 8.2±0.4 | 7.9±0.4 | 2.7±0.2 |
| $\boldsymbol{A_R}$ **[1/s$^2$] × 10$^8$** | 6.9±0.3 | 8.5±0.3 | 8.8±0.3 |
| $\boldsymbol{\tau_R}$ **[s] × 10$^{-9}$** | 2.6±0.2 | 2.8±0.2 | 3.1±0.3 |

**Sensitivity Analysis of Rotational Correlation Time Estimates**

To extract rotational correlation times ($\tau_c$) from the experimental $^{13}$C $T_1$ data at 25 °C, we used the Solomon–Bloembergen approximation in the fast-motion limit ($\omega_C\,\tau_c \ll 1$), where $1/T_1 \propto \tau_c$. However, as noted in the main text, the temperature-dependent profiles for the high-viscosity samples (e.g., 33 mol % ChCl) exhibit significant curvature at lower temperatures, suggesting that the system approaches the intermediate motional regime ($\omega_C\,\tau_c \approx 0.1$–$0.5$) rather than the extreme narrowing limit.

To ensure that the site-specific trends reported in Figure 8(i) are robust against deviations from the fast-motion assumption, we performed a sensitivity analysis using the full Bloembergen–Purcell–Pound (BPP) spectral density expression:

$$\frac{1}{T_1} = K\left[\frac{\tau_c}{1+(\omega_H-\omega_C)^2\tau_c^2} + \frac{3\tau_c}{1+\omega_C^2\tau_c^2} + \frac{6\tau_c}{1+(\omega_H+\omega_C)^2\tau_c^2}\right]$$

We solved this equation numerically for $\tau_c$ for a limiting case in which the measured $T_1$ is assumed to be near the minimum ($T_1 \approx 1.2\cdot T_{1,\min}$), which represents a maximal deviation from

the linear fast-motion approximation. This analysis shows that, while the absolute values of $\tau_c$ increase by approximately 15–25 % when nonlinear spectral-density effects are included for the 33 mol % sample, the relative hierarchy of segmental dynamics remains unchanged: $\tau_c$(NMe) < $\tau_c$(EG) < $\tau_c$($CH_2$–N).

Furthermore, the qualitative "plateau" behavior of the EG backbone up to 20 mol % remains distinct from the more monotonic slowing of the choline segments. We therefore conclude that the site-specific dynamical contrasts described in the text are physically meaningful and are not artifacts of the fast-motion approximation.

## NOESY Spectra

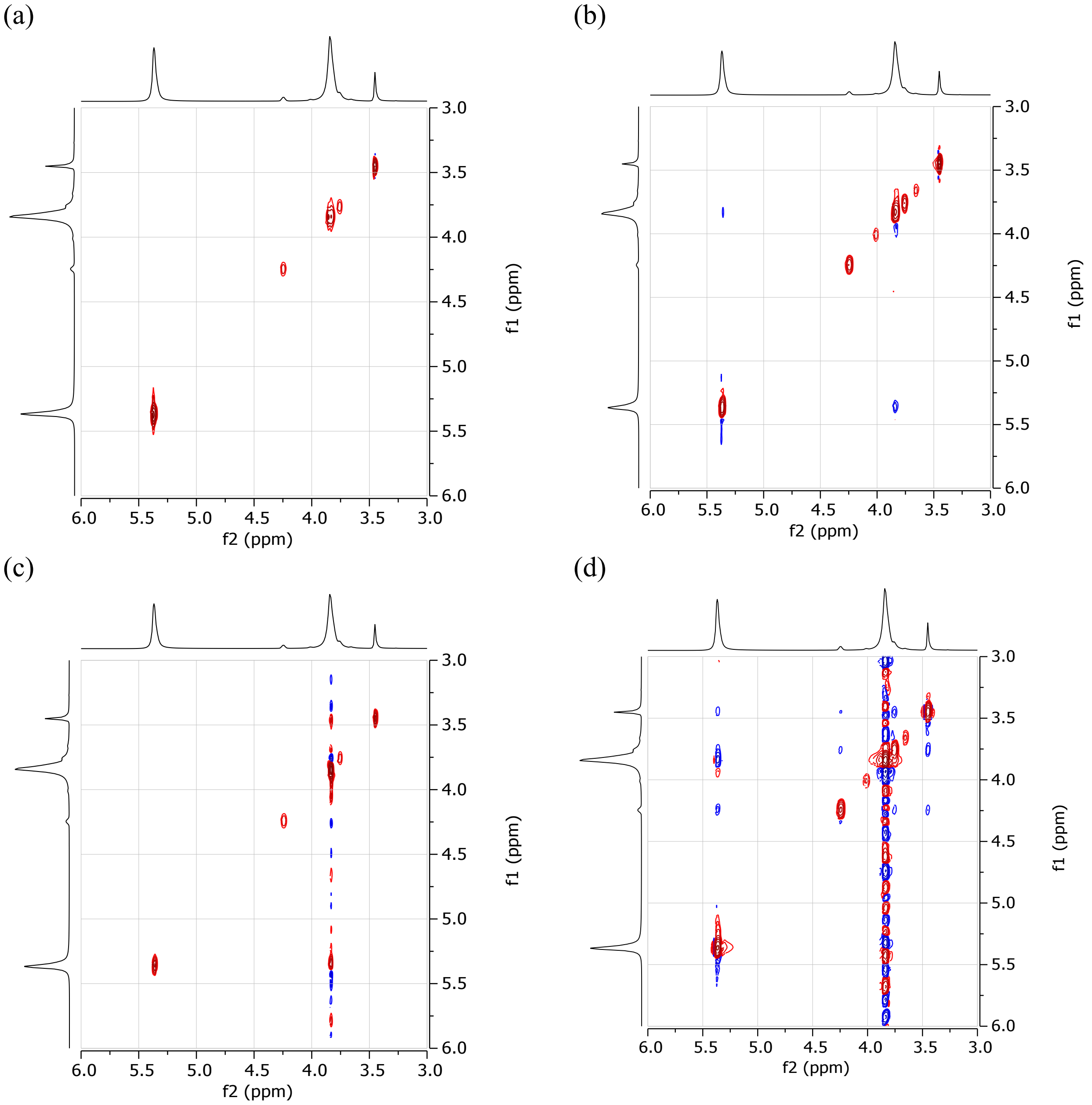


**Figure S6.** 2D NOESY spectra for 5 mol% ChCl in EG at 25 °C for four different mixing times (a) 50 ms, (b) 200 ms, (c) 400 ms, and (d) 800 ms (substantial $t_1$ noise).

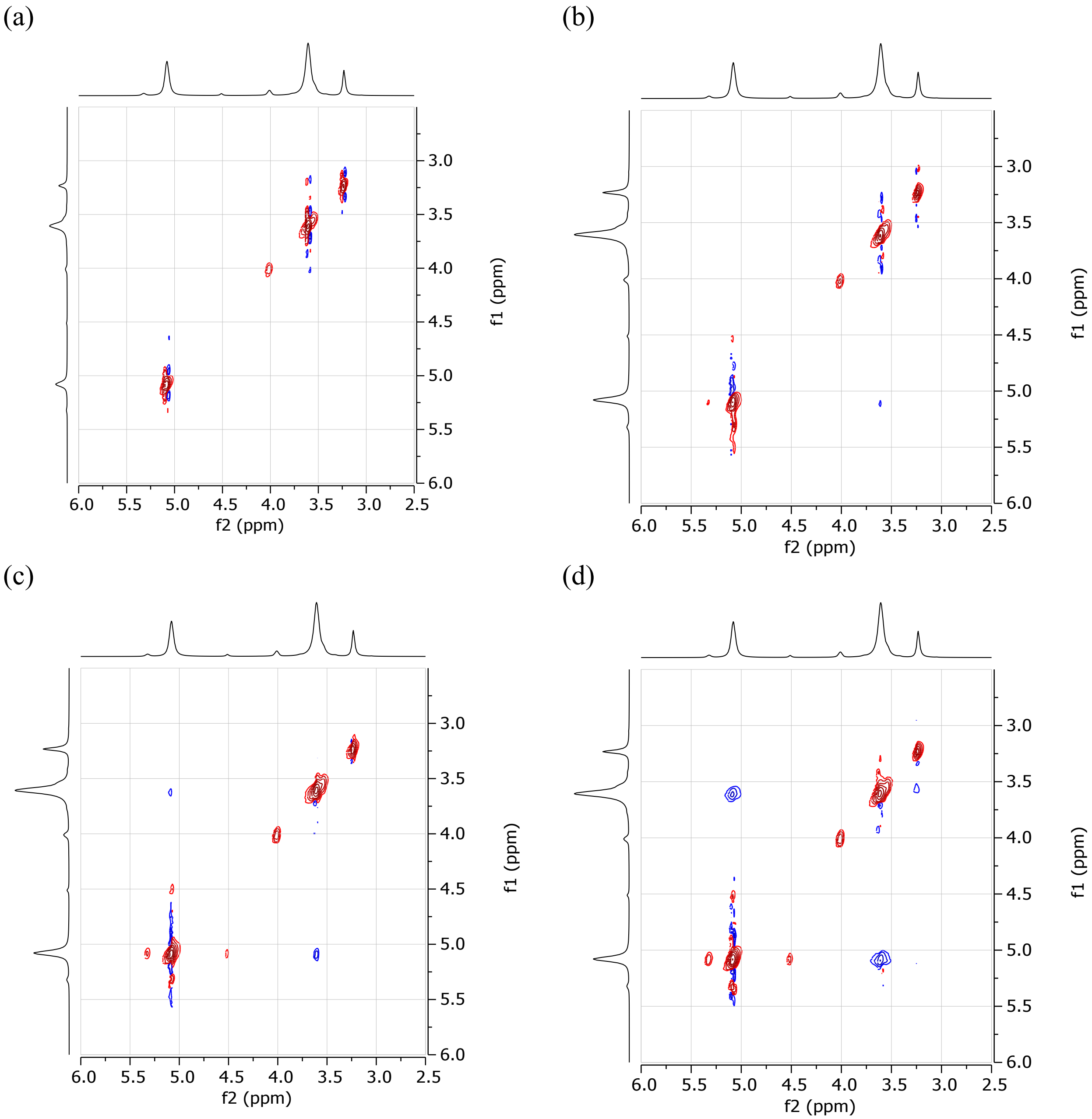


**Figure S7.** 2D NOESY spectra for 10 mol% ChCl in EG at 25 °C for four different mixing times (a) 50 ms, (b) 200 ms, (c) 400 ms, and (d) 800 ms.

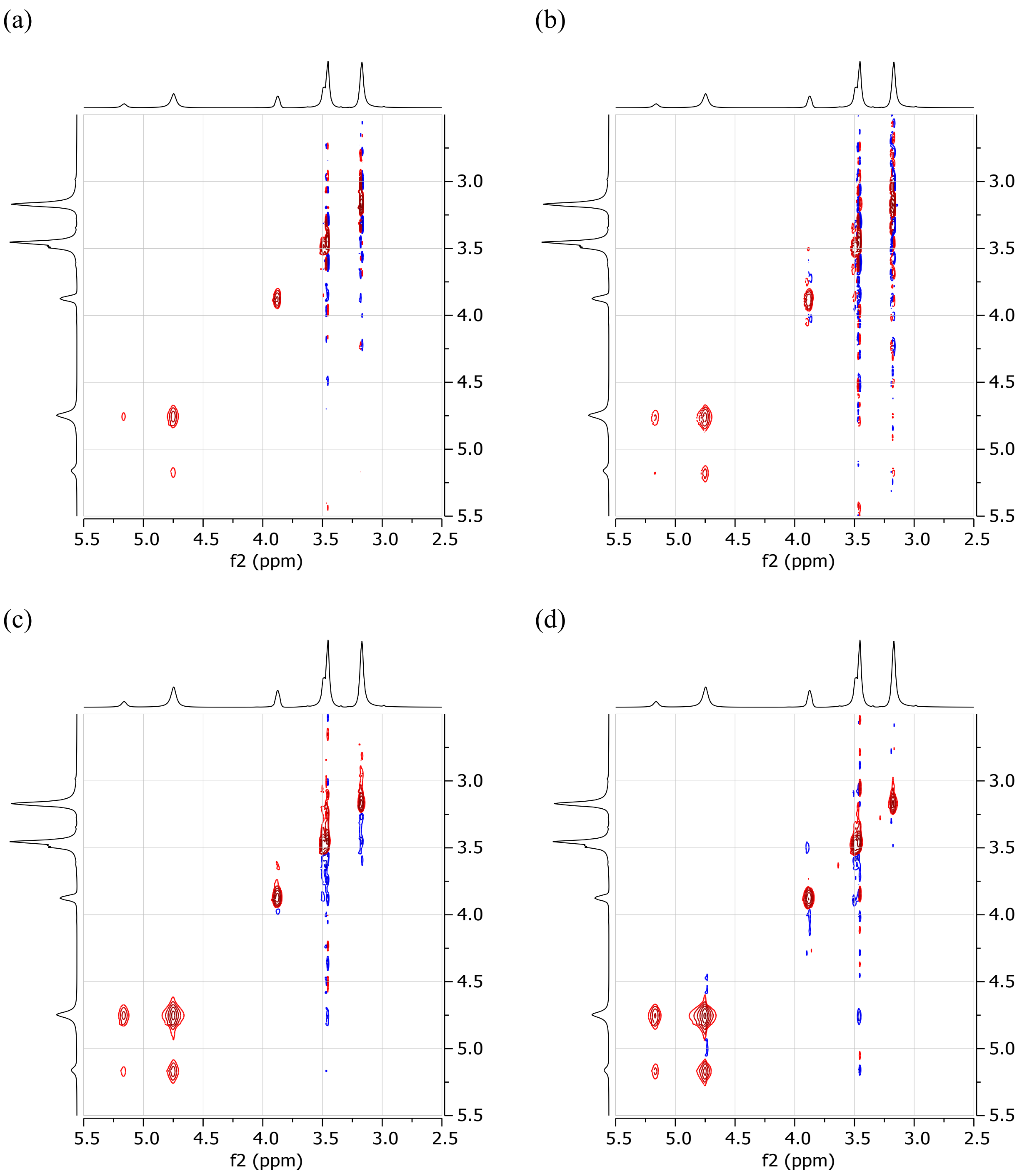


**Figure S8.** 2D NOESY spectra for 33 mol% ChCl in EG at 25 °C for four different mixing times (a) 50 ms, (b) 200 ms, (c) 400 ms, and (d) 800 ms.
